# Developing Sustainable Classification of Diseases via Deep Learning and Semi-Supervised Learning

**DOI:** 10.3390/healthcare8030291

**Published:** 2020-08-24

**Authors:** Chunwu Yin, Zhanbo Chen

**Affiliations:** 1 School of Information and Control Engineering, Xi’an University of Architecture and Technology, Xi’an 710055, China; chunwuy@hotmail.com; 2 School of Information and Statistics, Guangxi University of Finance and Economics, Nanning 530003, China; 3 Center of Guangxi Cooperative Innovation for Education Performance Assessment, Guangxi University of Finance and Economics, Nanning 530003, China

**Keywords:** deep learning, semi-supervised learning, self-training, disease classification

## Abstract

Disease classification based on machine learning has become a crucial research topic in the fields of genetics and molecular biology. Generally, disease classification involves a supervised learning style; i.e., it requires a large number of labelled samples to achieve good classification performance. However, in the majority of the cases, labelled samples are hard to obtain, so the amount of training data are limited. However, many unclassified (unlabelled) sequences have been deposited in public databases, which may help the training procedure. This method is called semi-supervised learning and is very useful in many applications. Self-training can be implemented using high- to low-confidence samples to prevent noisy samples from affecting the robustness of semi-supervised learning in the training process. The deep forest method with the hyperparameter settings used in this paper can achieve excellent performance. Therefore, in this work, we propose a novel combined deep learning model and semi-supervised learning with self-training approach to improve the performance in disease classification, which utilizes unlabelled samples to update a mechanism designed to increase the number of high-confidence pseudo-labelled samples. The experimental results show that our proposed model can achieve good performance in disease classification and disease-causing gene identification.

## 1. Introduction

Recently, bioinformatics technologies have provided efficient ways to diagnose diseases, and machine learning methods applied in bioinformatics have achieved remarkable breakthroughs in the field of disease diagnosis [1]. Disease classification based on gene expression levels can efficiently distinguish disease-causing genes efficiently, so it has become an effective method in disease diagnosis and gene expression levels assessment for different conditions [2,3,4]. The combination of data preprocessing and machine learning is an essential approach that improves the performances of many computer-aided diagnosis applications [5,6], including for log-count normalized original data in linear modelling [7]. Many state-of-the-art biological methods have been developed for disease classification. For example, a multiple feature evaluation approach (MFEA) of a multi-agent system has been proposed to improve the diagnoses of Parkinson’s disease [8]. A high-quality sampling approach has been proposed for imbalanced cancer samples for pre-diagnosis [9]. Supervised discriminative sparse principal component analysis (SDSPCA) has been used to study the pathogenesis of diseases and gene selection [10].

However, disease classification using gene expression data also faces challenges because of the characteristic high dimensions and small sample sizes [11]. Generally, large quantities of unlabelled samples are contained in datasets, because whole-genome gene expression profiling is still too expensive to be used by typical academic labs to generate a compendium of gene expression for a large number of conditions [12]. To improve the classification performance, semi-supervised learning, an incremental learning technique, has been designed to utilize unlabelled samples to obtain more labelled data. Semi-supervised learning has achieved many successful applications, for example, the semi-supervised functional module detection method based on non-negative matrix factorization [13] and semi-supervised hidden Markov models for biological sequence analysis [14]. Moreover, self-training is a special semi-supervised learning method that can implement learning from high- to low-confidence samples [15]. For example, self-training subspace clustering with low-rank representation has been proposed for cancer classification based on gene expression data [16]. A self-training algorithm that had been assumed feasible only for prokaryotic genomes has now been developed for gene identification [17]. Moreover, common classifiers do not achieve satisfactory accuracy because the number of samples is much smaller than the number of genes in gene expression data. To tackle these problems, a classifier named the forest deep neural network (FDNN) has been developed to integrate a deep neural network architecture with a supervised forest feature detector in RNA-seq expression datasets [18]. In addition, cancer subtype classification with deep learning can be used for single sample prediction to facilitate clinical implementation of cancer molecular subtyping [19]. The deep forest (DF) model, a decision tree ensemble approach with a non-neural network style deep model, is used in this work because it has been shown to achieve good performance in many tasks [20]. Furthermore, the deep forest exploits two types of forests, i.e., random forests (RFs) and completely random tree forests, which help enhance the diversity. Motivated by the lack of relevant research, we attempt to exploit the deep forest method for semi-supervised learning in biological tasks.

Many regularization methods have been proposed to identify significant genes to achieve high-performance disease diagnosis. Regularization methods have recently attracted increased attention in gene selection and have become a key technique to prevent over-fitting [21]. For example, a popular regularization term, the $L_{1}$ penalty, i.e., the Least Absolute Shrinkage and Selection Operator (LASSO), can assign redundant coefficients to zero for gene selection and has been applied to high-dimensional data [22,23]. Research on disease-causing gene selection involving the extended LASSO includes identification of context-specific gene regulatory networks with gene expression modelling using LASSO [24] and inference of gene expression networks with a weighted LASSO [25]. Stable feature selection can avoid negative influences when new training samples are added or removed [26]. Therefore, we investigate stable LASSO regularization to identify disease-causing genes in disease classification. In this paper, we propose a combined deep forest and semi-supervised with self-training (DSST) method to diagnosis diseases. With deep forest as a base model, semi-supervised learning such as self-training provides more high-confidence labelled samples for deep forest training. Three types of disease datasets are applied to our proposed approach to assess its effectiveness and robustness.

The rest of this paper is structured as follows. Section 2 presents a literature review of the various studies applying machine learning to disease diagnosis, including deep forest and semi-supervised learning. Section 3 describes our method. Section 4 introduces the dataset. We discuss the results and performance of our approach in Section 5. Finally, conclusions are presented in Section 6.

## 2. Literature Review

Machine learning methods for disease diagnosis can be traced back to the 1990s [27]. Since then, various machine learning methods have been investigated and tested for cancer classification. A forward fuzzy cooperative coevolution technique proposed for breast cancer diagnosis has achieved the best accuracy [28]. A weighted naive Bayesian (NB) method to predict breast cancer status with high F1 score and accuracy has been presented [29]. Recently, deep learning has achieved great success in various fields such as disease diagnosis. A new neighbouring ensemble predictor (NEP) method coupled with deep learning has been proposed to accurately predict a detected nuclear class label before quantitatively analysing the tissue constituents in whole-slide images to better understand cancer [30]. The application of deep learning methods to medical images can potentially improve the diagnostic accuracy, with algorithms achieving areas under the curve (AUCs) of 0.994 [31]. However, the ideal parameters of deep neural networks methods are difficult to determine. The deep forest model implements a novel classifier based on decision tree ensembles that explore how to construct deep models based on non-differentiable modules. Such models offer guidance to improve the underlying theory of deep learning and generate a deep forest exhibiting these characteristics [32]. Moreover, the number of hyper-parameters is fewer than that of deep neural networks and the complexity of a model can be automatically determined via data correlation. Various experimental results show that the model performance is robust after the hyper-parameters are set. Such models can achieve excellent performance with the default settings, even if data from distinct domains are considered. Many studies of deep forest methods have been developed [33,34], and these methods have been successfully used in image retrieval [35], and cancer subtype classification [36].

Semi-supervised learning, an active research topic in machine learning in recent years, aims to label an amount of unlabelled data to improve the performance of a model. Many recent successful examples of semi-supervised learning in bioinformatics have been presented. For example, a semi-supervised network to solve the high-dimensional problem of identifying known essential disease-causing genes has been proposed [37]. Chai et al. proposed a semi-supervised learning method with the Cox proportional hazard and accelerated failure time (AFT) models to predict disease survival time, and the performance of the model exceeded that of the Cox or AFT model alone [38]. Moreover, self-training, a type of semi-supervised learning, to learn by gradually including high- to low-confidence samples as pseudo-labelled samples has been proposed [39]. Self-training has been successfully applied to computer vision [40], data density peaks [41], computed tomography (CT) colonography [42] and other fields. In this paper, self-training with deep forest as base learners is used to learn from both labelled and unlabelled instances; in particular, the experiments shows that an ensemble learner provides additional improvement over the performance of adapted learners [43].

From a biological point of view, most likely only a few genes can strongly indicate targeted diseases, and most genes are irrelevant to cancer classification. The irrelevant genes may introduce noise and reduce the classification accuracy. Given the importance of these problems, effective gene selection methods can help classify different types of cancer and improve the prediction accuracy [44]. Stability selection provides an approach to avoid many false positives in biomarker recognition by repeatedly subsampling the data and only treating those variables assumed as biomarkers that are always important [45]. LASSO, as a primary variable selection method, is a popular regularization method and shrinks the regression coefficients towards zeros if their corresponding variables are not related to the model prediction target [46]. To obtain more sparse solutions, the $L_{p}$ norm is proposed, which simply consists of replacing the $L_{1}$ norm with the non-convex $L_{p}$ norm (0 < *p* < 1) [47]. A multi-stage convex relaxation scheme with a smoothed $L_{1}$ regularization is presented to solve problems with non-convex objective functions [48]. Zeng et al. [49] investigated the properties of the $L_{p}$ (0 < *p* < 1) penalties and revealed the extreme importance and special role of the $L_{1/2}$ regularization. Zou and Hastie [50] indicated that the $L_{p}$ (0 < *p* < 1) penalty can provide a different sparsity evaluation and that the $L_{q}$ (1 < *q* < 2) penalty can provide a grouping effect with different *q* values.

## 3. Methods

### 3.1. Semi-Supervised Learning with Deep Forest

**Figure 1:**
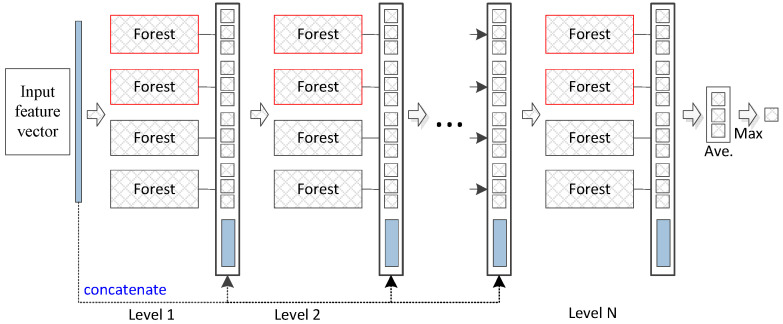
Diagram of the deep forest structure. Each level of the cascade consists of two random forests (red) and two completely random forests (black). Different coloured random forests represent different classes.

The deep forest approach provides an alternative to deep neural networks (DNNs) to learn super-hierarchical representations at low cost. Figure 1 illustrates the basic architecture of the deep forest model. The deep forest approach learns class distribution features directly based on multiple decision trees instead of learning via the hidden layers of DNNs. Additionally, an ensemble of forests can achieve more precise classification of distribution features since the random forest has a potent classification ability. We use previously reported parameter settings [20] to iteratively process the data in the experiments. In our proposed method, the convergence condition is that the training samples (combined original training and pseudo-labelled samples) achieve the best accuracy by employing the obtained pseudo-labelled samples ${\left(x\right)}_{i}^{n}$. In particular, labelled samples are used to train a base model to label unlabelled samples. Combined labelled and pseudo-labelled samples can then achieve higher performances in gene selection. The deep forest functions as a base model and is similar to the random forest ensemble model. In this paper, high-confidence samples are defined as those with smaller loss values; for example, the closer the *y* value is closer to 0 or 1 for the logistics regression, the smaller the loss value. These values represent high-confidence samples.

### 3.2. Self-Training

Consider a pseudo-labelled training dataset $D={\left(X_{i},y_{i}\right)}_{i=1}^{m}$ and a pseudo-labelled training dataset $D^{\prime }={\left(X_{i},y_{i}\right)}_{i=m+1}^{n}$ with *n* samples, where $X_{i}\in R^{d}$ is the $i^{th}$ sample and $y_{i=m+1}^{n}$ is the pseudo-label information according to the training of $X_{i}$ in a classification model. $f(X_{i},w)$ is a learned model, and *w* is a model parameter. $L(y_{i=m+1}^{n},f\left(X_{i=m+1}^{n},w\right))$ is a loss function of the $i^{th}$ sample. The objective of self-training is to simultaneously optimize the model parameter *w* and latent sample weights $v=[v_{m+1},v_{m+2},...,v_{n}]$ via a minimization Equation (1).

(1) $$\min_{w,v}E\left(w,v;\lambda \right)=\sum_{i=1}^{n}v_{i}L\left(y_{i}^{\prime },f\left(X_{i},w\right)\right)+g\left(\lambda ,v_{i}\right),$$

where $y^{\prime }$, $g(\lambda ,v_{i})$ and λ are pseudo-labels of unlabelled data, the self-training regularizer and a penalty that controls the learning pace, respectively. In general, given sample weights *v*, the minimization over *w* is a weighted loss minimization problem, independent of regularizer g(λ,v). If $g\left(\lambda ,v_{i}\right)=-\lambda v_{i}$, the optimal $v_{i}^{*}$ is calculated by

(2) $$v_{i}^{*}=\{\begin{array}{ccc} 1 & \;\;\; & if\;\;L(y_{i}^{\prime },f\left(X_{i},w\right))\leq \lambda \\ 0 & \; & otherwise \end{array}$$

## 4. Datasets

In this study, three public cancer datasets from the National Center for Biotechnology Information, U.S. National Library of Medicine (https://www.ncbi.nlm.nih.gov/geo)are utilized. A brief description of these datasets is shown in Table 1.

### 4.1. Lung Dataset

The lung cancer dataset (GSE4115) (https://www.ncbi.nlm.nih.gov/geo/query/acc.cgi?acc=GSE4115) is from Boston University Medical Center. The numbers of lung cancer and healthy samples are 97 and 90, respectively, and each sample contains 22,215 genes.

### 4.2. Breast Dataset

The breast cancer dataset (GSE21050) (https://www.ncbi.nlm.nih.gov/geo/query/acc.cgi?acc=GSE21050) from the French Institut Bergonie contains 310 samples, which consist of 183 lung cancer and 127 normal lung samples, with 54,677 genes as the model input.

### 4.3. Prostate Dataset

The prostate cancer dataset (https://www.ncbi.nlm.nih.gov/pmc/articles/PMC1524991/) is from the MIT Whitehead Institute. After preprocessing, the prostate dataset contains 102 samples and 12,600 genes in two classes, tumour and normal, which account for 52 and 50 samples, respectively.

## 5. Results

Three common methods are used for comparison to assess the performance of our approach: deep neural networks (DNNs), logistic regression (LR), support vector machine (SVM) and random forest (RF). In the experiments, a portion of the three disease datasets is treated as unlabelled samples to assess the classification accuracy of the proposed method. The labelled and unlabelled samples are randomly selected in every run of the program. Table 2 provides more details about the distributions of the datasets used in the experiments. The methodology of the tests encompasses 10-fold cross-validation to evaluate the learning of the methods and track the variation in their performance.

**Figure 2:**
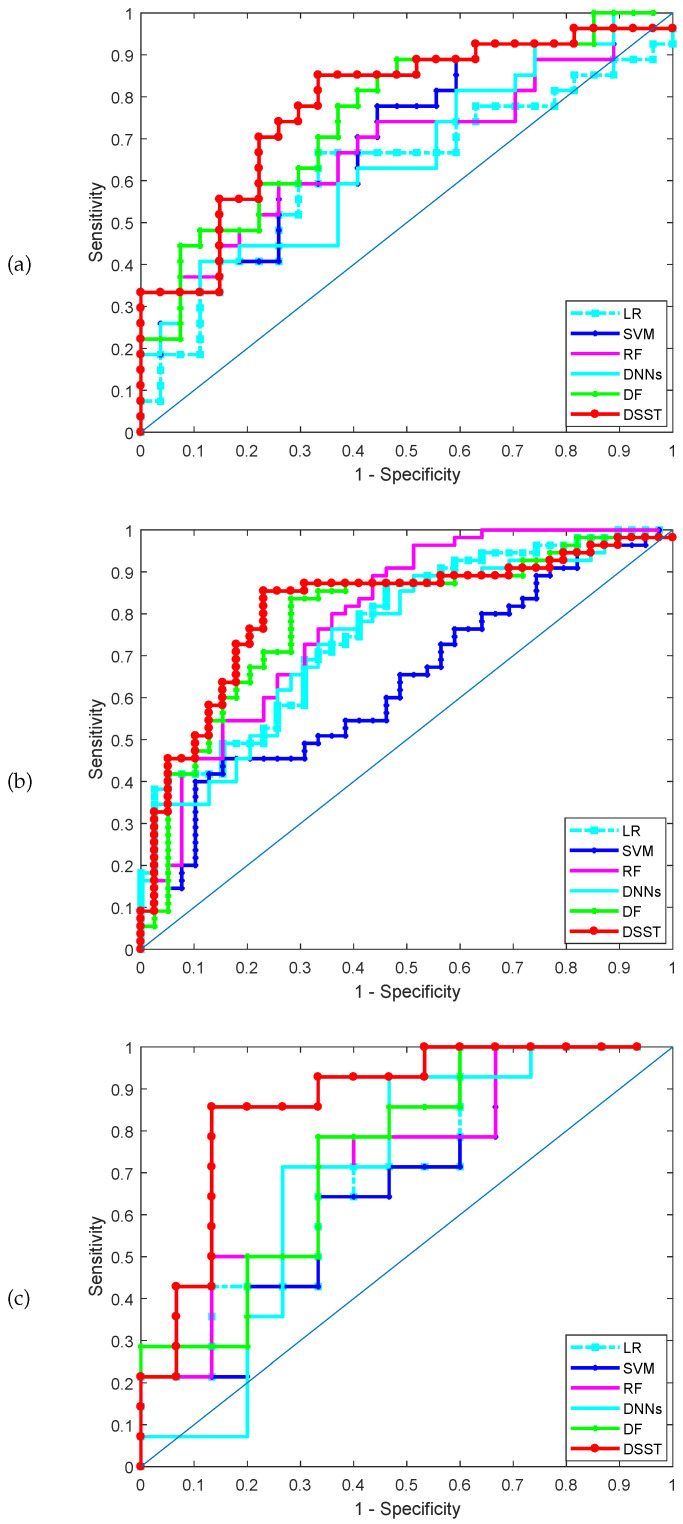
AUC-ROC in the three datasets, (**a**) GSE4115 (lung cancer), (**b**) GSE21050 (breast cancer), (**c**) prostate cancer.

The classification performance achieved by the various methods for the three datasets is shown in Table 3. Table 3 shows the results on the test set obtained by the five methods. DSST produces the best results. For example, for the lung cancer dataset (GSE4115), the DSST and deep forest (DF) rank first and second, respectively: the accuracy of DSST is 0.7389, which is higher than the values of 0.6618 and 0.5926 achieved by DF and RF, respectively. The receiver operating characteristic (ROC) curves obtained by the various methods in one run for the three datasets are shown in Figure 2, and the corresponding AUCs are shown in Table 3. DSST outperforms the other classifiers and the deep forest model. Moreover, DSST is characterized by greater sparsity than DF and the other models. Clearly, the F1 score of the DSST model is the highest; i.e., the robustness of the model is better than that of the remaining methods, which indicates that the mechanism used to update the pseudo-labelled samples is a crucial improvement for supervised learning model training.

### Discussion

To further illustrate the performance of our method in computer-aided diagnosis, stable LASSO is used in this work [45]. The top-10 ranked genes selected by stable LASSO in the various datasets are listed in Table 4, Table 5 and Table 6. Most stability scores are close to 1, which indicates that the selected genes are robust. Additionally, the *p*-values indicate that the results are significant. Many studies consider function analysis for gene expression. For example, USP6NL in Table 4 acts as a GTPase-activating protein for RAB5A [51]. LMX1A in Table 5 acts as a tumor suppressor to inhibit cancer cell progression [52]. TP63 in Table 6 encodes a member of the p53 family of transcription factors, in which the functional domains of p53 family proteins include an N-terminal transactivation domain, a central DNA-binding domain and an oligomerization domain [53].

**Figure 3:**
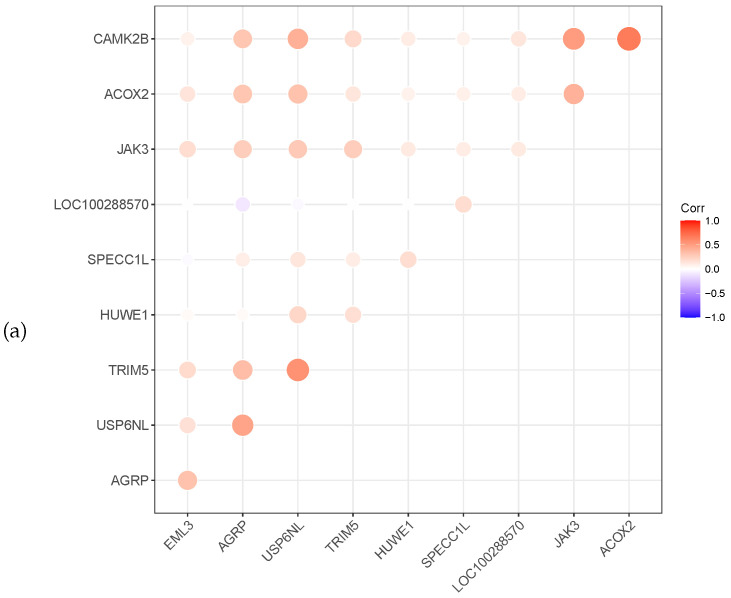

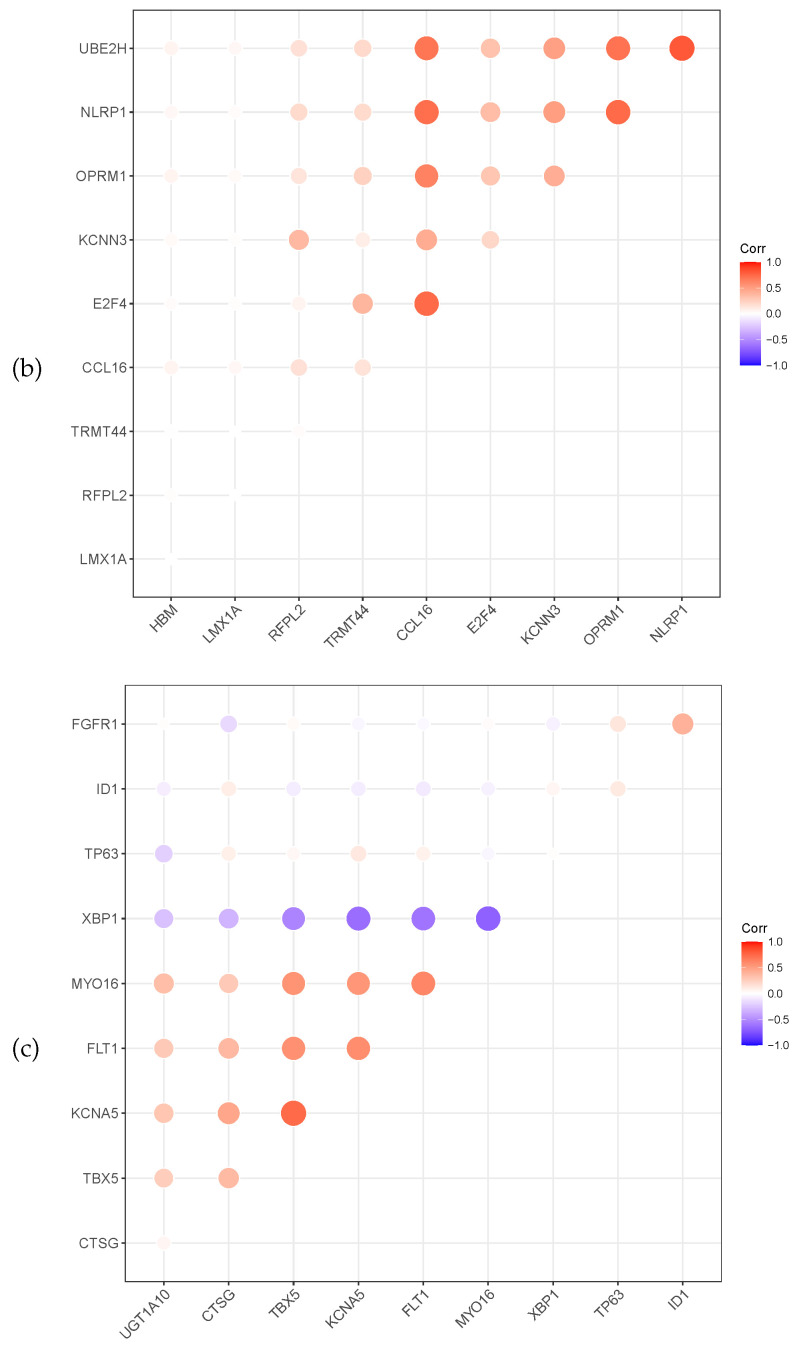
Relevance display by heat map for the three datasets, (**a**) GSE4115 (lung cancer), (**b**) GSE21050 (breast cancer) and (**c**) prostate cancer.

Meanwhile, the heat map correlation between the genes is illustrated in Figure 3. A red colour indicates a positive correlation, while a violet colour indicates a negative correlation. The darker the colour, the stronger the correlation. Figure 3 shows that most selected genes have a positive correlation. The gene XBP1 of prostate cancer is negatively correlated with the other six genes.

## 6. Conclusions

In this paper, we proposed deep forest and semi-supervised with self-training (called DSST) to solve disease classification and gene selection problem based on different types of diseases. The deep forest method is consistently superior to other conventional classification methods, possibly because the deep forest approach learns more significant advanced features in the learning process. Semi-supervised learning provides an effective alternative to alleviate the challenges of over-fitting and improves the robustness of the model in the experimental process. Improved experimental results can be obtained by combining semi-supervised learning and the deep forest model. By simultaneously considering all classes during the gene selection stages, our proposed extensions identify genes leading to more accurate computer-aided diagnosis by doctors.

In the experiments, we used datasets for three types of diseases to assess and investigate the performance of our method using trained from 10-fold cross-validation and different sizes datasets. The results show that our proposed disease classification approach has achieved higher prediction accuracy than other methods published in the literature. However, the relevance threshold is different in the context of classification performance when the number of training instances is small. Therefore, how to determine the relevance threshold in the adaptive problem will be a focus of our work in the future. Additionally, we believe that our mechanism can also be applied to other types of disease diagnosis problems and can be expanded to various classifications of disease states.

## Figures and Tables

**Table 1:** Three publicly available disease datasets.

Dataset	Disease Type	No. of Samples	No. of Genes	Microarray Platform	Class
1	Lung	187	22,215	Affymetrix Human Genome U133A Array	Normal/Tumour
2	Breast	310	54,677	Affymetrix Human Genome U133 Plus 2.0 Array	Normal/Tumour
3	Prostate	102	12,600	Hybridization to U95Av2 arrays	Normal/Tumour

**Table 2:** Details of the experimental dataset settings.

Dataset	Disease Type	Labelled Samples	Unlabelled Samples	Testing Samples	No. of Genes
1	Lung	65	65	57	22,215
2	Breast	109	109	92	54,677
3	Prostate	36	36	30	12,600

**Table 3:** Performance comparison of various models.

Dataset	Model	Accuracy	AUC	Recall	Precision	F1-Score
Lung cancer	LR	0.5926	0.5885	0.4074	0.6471	0.5000
	SVM	0.6481	0.6406	0.4815	0.7222	0.5778
	RF	0.5926	0.6036	0.8148	0.5641	0.6667
	DNNs	0.6023	0.6173	0.5555	0.9259	0.6944
	DF	0.6618	0.6708	0.7037	0.6333	0.6667
	DSST	0.7389	0.7209	0.7778	0.7000	0.7368
Breast cancer	LR	0.7128	0.7091	0.8909	0.7000	0.7840
	SVM	0.5957	0.5921	0.9818	0.5934	0.7397
	RF	0.7447	0.7245	0.9818	0.7013	0.8182
	DNNs	0.7021	0.7170	0.7636	0.7368	0.7500
	DF	0.7766	0.7702	0.8182	0.8036	0.8108
	DSST	0.8085	0.8093	0.8545	0.8246	0.8393
Prostate cancer	LR	0.6333	0.6429	0.6667	0.6250	0.6452
	SVM	0.5862	0.6381	0.6667	0.5882	0.6250
	RF	0.6552	0.6762	0.7333	0.6471	0.6875
	DNNs	0.6333	0.6286	0.7333	0.6111	0.6667
	DF	0.6897	0.7238	0.8000	0.6667	0.7273
	DSST	0.7931	0.7857	0.7333	0.8462	0.7857

**Table 4:** The top-10 ranked informative genes found in the lung cancer dataset based on stable Least Absolute Shrinkage and Selection Operator (LASSO).

Gene Name	Gene Symbol	Stable Score	*p*-Value
USP6 N-terminal like	(USP6NL)	1	<0.01
acyl-CoA oxidase 2	(ACOX2)	0.98	<0.01
agouti related neuropeptide	(AGRP)	0.53	<0.01
HECT, UBA and WWE domain containing 1, E3 ubiquitin protein ligase	(HUWE1)	0.99	<0.01
calcium/calmodulin dependent protein kinase II beta	(CAMK2B)	1	<0.01
tripartite motif containing 5	(TRIM5)	1	<0.01
Janus kinase 3	(JAK3)	1	<0.01
sperm antigen with calponin homology and coiled-coil domains 1 like	(SPECC1L)	0.96	<0.01
echinoderm microtubule associated protein like 3	(EML3)	1	<0.01
glycosylphosphatidylinositol anchor attachment protein 1 homolog (yeast) pseudogene	(LOC100288570)	1	<0.01

**Table 5:** The top-10 ranked informative genes found in the breast cancer dataset based on stable LASSO.

Gene Name	Gene Symbol	Stable Score	*p*-Value
LIM homeobox transcription factor 1 alpha	(LMX1A)	0.96	<0.01
tRNA methyltransferase 44 homolog (S. cerevisiae)	(TRMT44)	0.95	<0.01
NLR family pyrin domain containing 1	(NLRP1)	0.69	<0.01
ret finger protein like 2	(RFPL2)	1	<0.01
C-C motif chemokine ligand 16	(CCL16)	0.92	<0.01
opioid receptor mu 1	(OPRM1)	0.7	<0.01
ubiquitin conjugating enzyme E2 H	(UBE2H)	0.81	<0.01
potassium calcium-activated channel subfamily N member 3	(KCNN3)	0.98	<0.01
haemoglobin subunit mu	(HBM)	1	<0.01
E2F transcription factor 4	(E2F4)	1	<0.01

**Table 6:** The top-10 ranked informative genes found in the prostate cancer dataset based on stable LASSO.

Gene Name	Gene Symbol	Stable Score	*p*-Value
fms related tyrosine kinase 1	(FLT1)	0	<0.01
tumour protein p63	(TP63)	0.56	<0.01
UDP glucuronosyltransferase family 1 member A10	(UGT1A10)	0.73	<0.01
T-box 5	(TBX5)	1	<0.01
potassium voltage-gated channel subfamily A member 5	(KCNA5)	1	<0.01
myosin XVI	(MYO16)	0.83	<0.01
inhibitor of DNA binding 1, HLH protein	(ID1)	0.86	<0.01
cathepsin G	(CTSG)	1	<0.01
X-box binding protein 1	(XBP1)	0.95	<0.01
fibroblast growth factor receptor 1	(FGFR1)	1	<0.01
